# Acceptability of a Newly Developed Financial Program for Caregivers of Individuals Living with a Chronic Illness

**DOI:** 10.1093/geroni/igab046.3658

**Published:** 2021-12-17

**Authors:** Claire Grant, Sam Fazio, Monica Moreno, Beth Kallmyer, Kerry Lanigan, Katherine Judge

**Affiliations:** 1 Cleveland State University, South Euclid, Ohio, United States; 2 Alzheimer's Association, Chicago, Illinois, United States; 3 Cleveland State University, Cleveland, Ohio, United States

## Abstract

Over 40 million informal caregivers provide care to adults aged 50 or older with a chronic illness. In addition to the negative health and well-being impact, caregivers experience financial difficulties including lost income, retirement benefits, future earnings, and unanticipated out-of-pocket costs. Few evidence-informed programs exist to assist caregivers in understanding and managing these financial tasks. This poster presents preliminary acceptability data from 71 caregivers who completed the newly developed Managing Money: A Caregiver’s Guide to Finances program. The 60-minute program was delivered online by trained community educators and addressed caregiving costs/impacts; future planning; initiating conversations; avoiding financial abuse/fraud; and identifying needs. Participants (Mage = 59.45, SD = 11.31) were 87.32% female with 77.46% self-identified as White, 9.86%, as Black, and 8.45% as Hispanic/Latino. Using a Likert scale (1=strongly disagree to 5=strongly agree), participants indicated the program: 1) identified financial challenges (M= 4.11 ; SD = 0.73); 2) provided information for managing money (M = 3.99; SD = 0.64); 3) content was easy to read/understand (M = 4.49; SD = 0.61); 4) program length was appropriate (M = 4.18; SD= 0.68); and 5) activities were helpful (M = 4.13; SD = 0.69). Participants rated the program as very important (M = 4.54; SD = 0.82) and would highly recommend (M = 4.52; SD = 0.53). Results indicated the program was well-received by participants and highly acceptable. Discussion will highlight key program features designed to promote acceptability along with the importance of measuring acceptability for large-scale implementation along with next research steps.

